# Acute Scrotal Pain and Swelling as an Atypical Presentation of Sweet’s Syndrome

**DOI:** 10.7759/cureus.87218

**Published:** 2025-07-03

**Authors:** Jeffrey Jiang, An Huynh, Angel Wu, Alexander Nirenberg, Weranja Ranasinghe

**Affiliations:** 1 Department of Surgery, Monash Health, Melbourne, AUS; 2 Department of Urology, Monash Health, Melbourne, AUS; 3 Department of Diagnostic Imaging, Monash Health, Melbourne, AUS; 4 Department of Pathology, Australasian College of Cutaneous Oncology, Melbourne, AUS

**Keywords:** neutrophilic dermatosis, scrotal pain, scrotal swelling, sweet's syndrome, urology consults

## Abstract

We present a unique case of Sweet’s syndrome associated with scrotal pain and swelling in a healthy male with no rheumatological or malignant risk factors. In the absence of typical urological presentations, diagnostic challenges included ruling out surgical emergencies in addition to skin biopsy and commencement of steroid treatment. This case highlights the importance of comprehensive examination and investigation to prevent surgical complications and consideration of rheumatological pathologies in atypical scrotal presentations.

## Introduction

Sweet’s syndrome, also known as acute febrile neutrophilic dermatosis, is a rare condition named after Dr. Robert Douglas Sweet in 1964 [1]. Sweet’s syndrome typically presents in the setting of infection, inflammatory bowel disease, pregnancy, malignancy, or drug reaction [2]. Clinical symptoms include fever, musculoskeletal pain, and ocular manifestations. The rash associated with Sweet’s syndrome can range from papules and vesicles to plaques and nodules with a red and purple colour change [3]. The rash can be located all over the body but is most frequently located on the upper extremities. Laboratory investigations typically demonstrate neutrophilia and elevated erythrocyte sedimentation rate (ESR). Systemic corticosteroids are the mainstay treatment for Sweet’s syndrome [3]. While cutaneous and systemic manifestations are well described, genital or scrotal involvement is exceptionally rare, with very few cases reported in the literature. This case presents a novel and diagnostically challenging manifestation of Sweet’s syndrome, highlighting the importance of recognizing atypical presentations to avoid unnecessary surgical intervention.

## Case presentation

A male in his 30s presented to the emergency department with painful purpura and bilateral testicular pain and swelling following a recent viral respiratory tract infection, for which he was prescribed oral amoxicillin in the community. His past medical history includes asthma. He had no recent travel or sick contacts. He has a stable long-term partner and no history of intravenous drug use. The purpuric rash was most prominent on the lower limbs.

**Figure 1:**
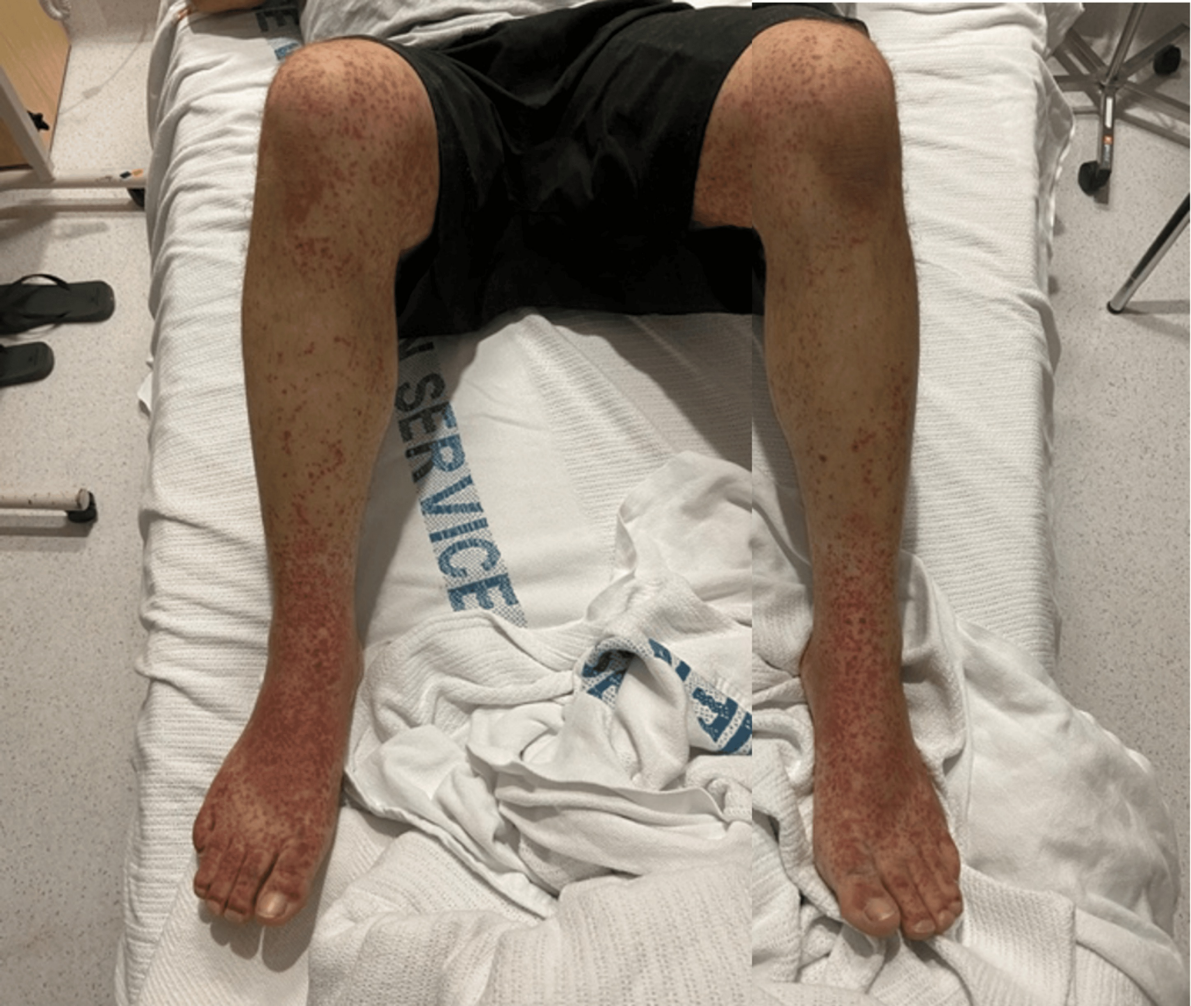
Bilateral lower limb purpuric rash

The rash was purpuric and most prominent on the lower limbs. There was associated distal lower limb oedema and intermittent arthralgia (Figure 1). Two days later, the patient complained of bilateral testicular pain and swelling. On examination, he was afebrile and hemodynamically stable. There was no abdominal tenderness, lymphadenopathy, or organomegaly. Scrotal examination revealed mild scrotal pain and mild-to-moderate scrotal oedema, worse on the right side, with no overlying skin changes present in the penoscrotal region. He had no ocular signs or symptoms.

Investigations

Initial blood tests showed a normal platelet count, clotting profile, liver function, and renal function. All inflammatory markers, including white cell count, C-reactive protein, and erythrocyte sedimentation rate, were within normal limits. On urinalysis, there was no proteinuria and no growth. Testicular cancer markers, alpha-fetoprotein, beta human chorionic gonadotropin, and lactate dehydrogenase, were all within normal limits.

**Figure 2:**
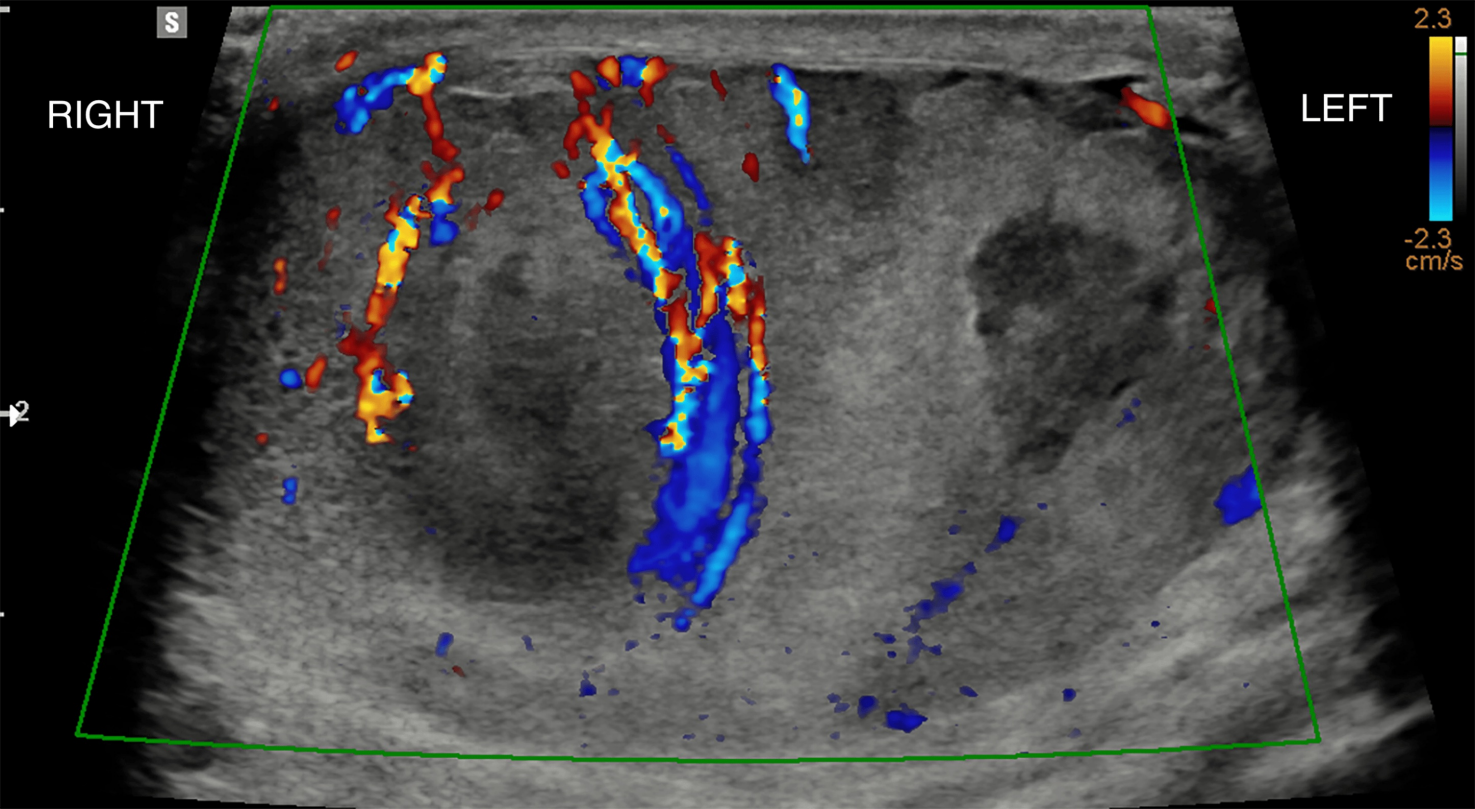
Scrotal ultrasound demonstrating hyperaemia of the testis and epididymis bilaterally Significantly worse on the right testis compared to the left testis.

**Figure 3:**
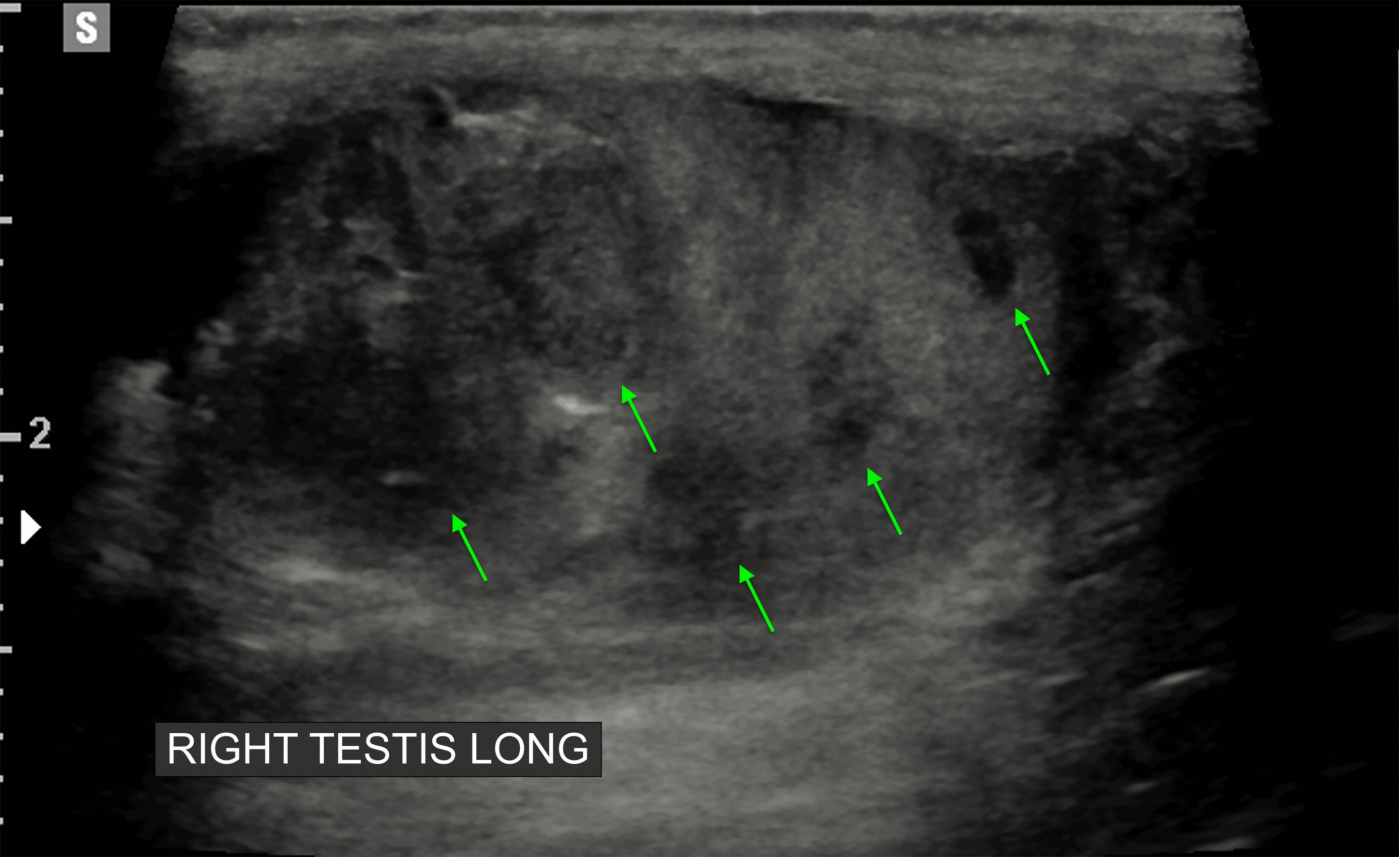
Right testes with multiple hypoechoic, avascular regions (demonstrated by green arrows)

Scrotal ultrasound reported hypoechoic regions with increased vascularity, suspicious for testicular abscesses on the right side, and mild bilateral hydroceles. However, these may have also presented inflammatory lesions (Figures 2-3).

**Figure 4:**
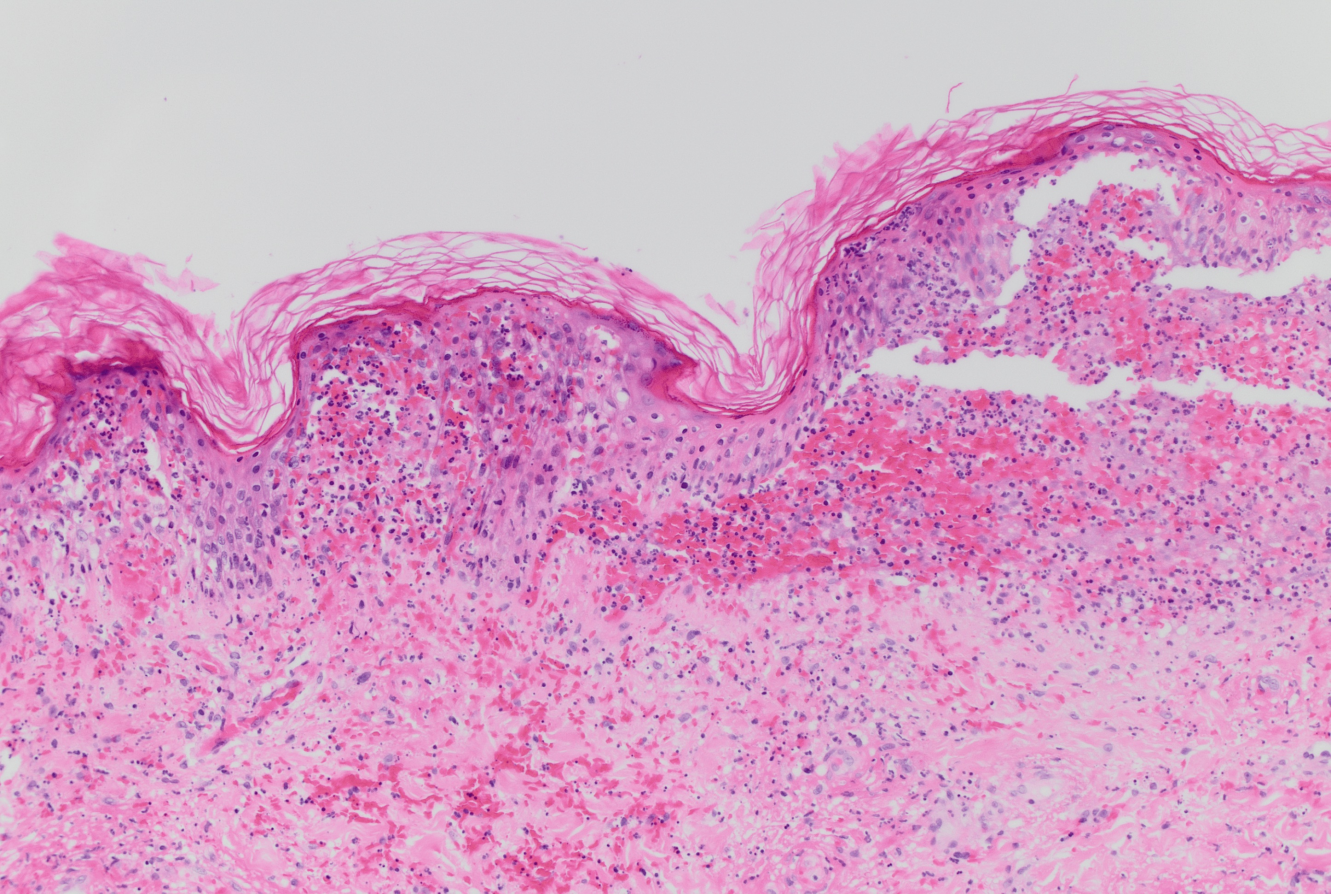
Right thigh skin punch biopsy with moderate interstitial and perivascular neutrophilic infiltrate abutting and extending into the epidermis (magnification 200x)

His immunoglobulin A (IgA) level was 3.9 g/L, and his skin punch biopsy revealed neutrophilic dermatosis consistent with Sweet’s syndrome (Figure 4).

The biopsy demonstrated a moderate interstitial and perivascular neutrophilic infiltrate that abuts and extends into the epidermis. There was no immunofluorescence staining for IgG, IgA, IgM, C3, or specific staining for C1q or fibrinogen, making IgA vasculitis unlikely.

Treatment

Given the presence of painful purpura, the patient was initially commenced on high-dose 50 mg oral prednisolone.

Following the ultrasound report suggestive of scrotal abscesses, the patient was commenced on broad-spectrum intravenous antibiotics; however, this was ceased one day later in the absence of any infective pathology. The patient responded well to prednisolone and was discharged on day three with a steroid weaning plan and follow-up in the rheumatology outpatient clinic in one month.

Outcome and follow-up

The patient was seen in the rheumatology vasculitis clinic four weeks after discharge, with improvement in pain symptoms. The purpuric rash of the lower abdomen and limbs was still present, and he was continued on oral prednisolone with further monitoring and follow up.

## Discussion

The unique aspect of this case is the presence of testicular pain and swelling associated with Sweet’s syndrome, with minimal previously documented reports in the literature. In 2020, a case reported by Ensley et al. [4] described a patient with known acute myelogenous leukaemia presenting with Sweet’s syndrome rash focused on the genital region. Given the location of the rash, the patient experienced secondary penile and scrotal pain. Another case in 2017 by Adamska et al. [5] described a patient with simultaneous Sweet’s syndrome and testicular discomfort on the background of previously undiagnosed episodes of acute rash, fever, and malaise. Given the absence of a precipitant, idiopathic epididymitis was attributed as the likely trigger for the patient’s episode. While both these cases describe scrotal association in Sweet’s syndrome, neither describes testicular pain and swelling as an additional symptom in the clinical picture of Sweet’s syndrome.

Testicular pain in the acute setting warrants an immediate surgical review to exclude torsion, trauma, and infective causes, such as epididymitis, orchitis, and abscess. In particular, testicular torsion and trauma often require time-sensitive intervention to prevent irreversible ischemia, and thus assessment should not be delayed.

In our case, the temporal correlation between the development of painful purpura and testicular swelling, the absence of infectious or malignant causes on investigation, and the clinical improvement with corticosteroid therapy supported a rheumatological diagnosis. Other features that vary from the typical presentation of Sweet’s syndrome include the absence of fevers, normal inflammatory markers, and the absence of neutrophilia. Sweet’s syndrome rash typically presents in the upper extremities, whereas this patient had predominantly a lower limb rash. Pathologically, there was a neutrophilic infiltrate into the epidermis, while typically this spares the epidermis in Sweet’s syndrome. The presentation highlights that Sweet’s syndrome should remain in the differential diagnosis even in the absence of typical systemic findings.

## Conclusions

In conclusion, our case documents scrotal pain and swelling as a clinical manifestation of Sweet’s syndrome. We recommend an immediate review of clinical signs and symptoms to rule out a surgical pathology. In light of other systemic symptoms, a comprehensive serology panel and ultrasound should be performed to look for an alternative diagnosis. Importantly, this case also highlights the diagnostic challenge posed by atypical presentations such as the absence of fever, neutrophilia, or elevated inflammatory markers, which may delay recognition. As such, clinicians should maintain a broad range of differentials in the absence of typical features. Early commencement of systemic steroids and rheumatological input is advisable where Sweet's syndrome is suspected.

## References

[1] Sweet RB (1964). An acute febrile neutrophilic dermatosis. Br J Dermatol.

[2] Rochet NM, Chavan RN, Cappel MA, Wada DA, Gibson LE (2013). Sweet syndrome: clinical presentation, associations, and response to treatment in 77 patients. J Am Acad Dermatol.

[3] Cohen PR (2007). Sweet's syndrome--a comprehensive review of an acute febrile neutrophilic dermatosis. Orphanet J Rare Dis.

[4] Ensley D, Evans GH (2020). Genital Sweet's syndrome in a patient with acute myelogenous leukemia. Urol Case Rep.

[5] Adamska U, Męcińska-Jundziłł K, Białecka A (2017). Sweet's syndrome with idiopathic epididymitis. Postepy Dermatol Alergol.

